# ChemODE: A Physics-Informed Neural Surrogate for Robust Pharmacokinetics and Biochemical Dynamics

**DOI:** 10.1021/acsomega.6c04487

**Published:** 2026-09-30

**Authors:** Wit Kulvutiroj

**Affiliations:** 520984 Kamnoetvidya Science Academy, Wang Chan District, Pa Yup Nai, Rayong 21210, Thailand

## Abstract

The automated discovery of governing kinetic laws from observational data is a critical challenge in systems biology, pharmacology, and chemical engineering. In these experimental domains, data-driven mechanism discovery is complicated by sparse sampling, measurement noise (e.g., blood assays), and the prevalence of nonlinear saturation limits (Michaelis-Menten kinetics). Existing symbolic regression methods, such as SINDy, rely on polynomial basis functions that fundamentally fail to extrapolate physical saturation, and their reliance on numerical differentiation degrades catastrophically in high-noise regimes (>5%). In this work, we introduce ChemODE, a physics-informed gray-box framework tailored for biochemical and pharmacokinetic (PK) networks. ChemODE integrates (1) Constrained Rational Layers to autonomously enforce physical saturation laws, (2) On-the-fly Feature Normalization to resolve magnitude bias in multicompartment scale separations, and (3) a noise-robust integral-based optimization scheme. We validate ChemODE on fundamental biological benchmarks, including the nonlinear Brusselator limit cycle and a synthetic 3-compartment pharmacokinetic model. To demonstrate clinical applicability, ChemODE successfully extracts physiological absorption and elimination rates from real-world human theophylline pharmacokinetic assays. Furthermore, rigorous uncertainty quantification (10-seed ensembles) proves that ChemODE recovers enzyme kinetics with high precision (*V*_max_ error ≈ 1%), significantly outperforming unconstrained baselines. This work establishes that integral-based minimization, combined with rational architectural constraints and a neural residual paradigm, provides a robust surrogate modeling path for interpretable mechanism discovery in noise-dominated biochemical settings.

## Graphical abstract

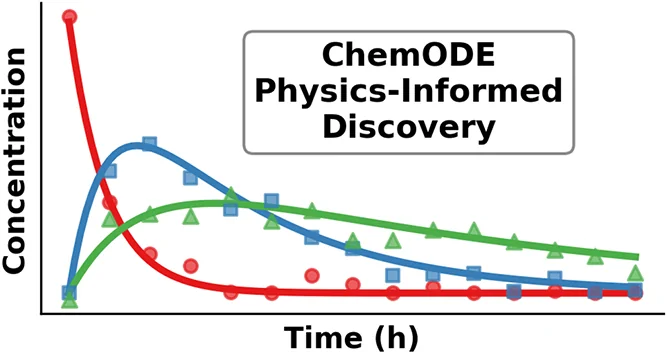

## 1 Introduction

Biochemical kinetics, the study of reaction rates, enzyme catalysis, and multicompartment drug distribution, is fundamental to disciplines ranging from pharmaceutical development to systems biology.^1^ The central goal of kinetic modeling is to determine a set of ordinary differential equations (ODEs) describing how species and drug concentrations evolve over time. Traditionally, this is a deductive process limited by human intuition, manual hypothesis testing, and standard compartmental fitting.

The emergence of scientific machine learning (SciML) has prompted a shift toward inductive data-driven discovery. Modern neural architectures, such as ChemNODE^2^ and ChemKAN,^3^ have demonstrated remarkable capabilities in handling multiscale chemical systems. However, these methods typically operate as high-dimensional black-box solvers, sacrificing the mechanistic interpretability required by pharmacologists and physical chemists. Conversely, symbolic regression methods, notably SINDy,^4^ frame the discovery process as selecting the sparsest subset of terms from a candidate library to yield highly interpretable equations.

Despite its computational efficiency, applying standard symbolic regression to biological systems presents two fundamental roadblocks. First, biological systems are dominated by enzyme catalysis and receptor binding, which follow saturation limits (e.g., Michaelis-Menten or Langmuir kinetics). Standard SINDy libraries rely on Taylor-expanded polynomials that cannot express true saturation and diverge wildly outside of the training domain. Second, SINDy relies on the approximation of time derivatives from the discrete data. As noted by Cortiella et al.,^5^ numerical differentiation inherently amplifies high-frequency noise. In typical biological assays, where noise exceeds 5%, derivative-based methods frequently yield physically impossible models.

Simultaneously, recent advancements in scientific machine learning have expanded the toolkit for dynamical systems. These include foundational Neural ODEs,^6^ physics-informed neural networks (PINNs),^7^ universal differential equations,^8^ and stiff Neural ODE formulations.^9^ Furthermore, these frameworks have very recently been adapted for complex physiologically based pharmacokinetic (PK) modeling and biomedical parameter inference.^10–12^ Parallel efforts in PDE discovery and symbolic regression have yielded robust variants such as DeepMoD,^13^ data-driven PDE finders,^14^ Weak SINDy,^15^ and implicit SINDy.^16^ Despite these advances, standard Neural ODEs typically operate as black-box models lacking structural interpretability. Conversely, while PINNs enforce physical laws via loss penalties, they often struggle with the stiff optimization landscapes inherent to biological data and require complex hyperparameter tuning to enforce hard constraints like enzyme saturation positivity.

In this work, we address these limitations with ChemODE, a physics-informed neural surrogate that is designed explicitly for biological and pharmacokinetic networks. We introduce constrained rational layers that natively model saturation fractions without violating physical positivity constraints. To address noisy sampling, ChemODE treats the derivative function as a neural network and integrates it directly over time, effectively acting as a physics-informed low-pass filter.^8^ By combining this integral formulation with on-the-fly feature normalization, we demonstrate that ChemODE successfully recovers governing biological motifs from sparse, noisy clinical data where standard polynomial and derivative-based baselines fail.

Fundamentally, ChemODE establishes a necessary interdisciplinary bridge by integrating principles from physics (enforcing mass conservation and positivity constraints), chemistry (learning kinetic rate laws), biochemistry (capturing enzyme saturation limits, such as CYP450-mediated clearance via Michaelis-Menten kinetics), and biostatistics (handling sparse, noise-dominated clinical assays through an integral-based loss function). By unifying these domains, we constructed a robust framework for identifying complex biochemical dynamics.

## 2 Methodology

ChemODE frames the kinetic discovery problem as a Neural ODE task where the derivative function *f*_θ_(*t*, **y**) is defined by a constrained symbolic layer rather than a black-box multi-layer perceptron. The core innovation lies in conditioning this optimization problem to handle the specific numerical pathologies of biological data: saturation limits, magnitude bias across different physiological compartments, and experimental noise.

### 2.1 Constrained Rational Layer (Saturation Kinetics)

Many biological and catalytic systems exhibit saturation (e.g., Michaelis-Menten enzyme clearance in the liver). Polynomial libraries typically fail to capture these behaviors, requiring deep Taylor-expansion approximations.

We extend the architecture to Rational Regression, modeling the time derivative as the quotient of two linear operators. To enforce physical plausibility specifically that the Michaelis constant (*K*_m_) must be strictly positive, we apply a Softplus constraint to the denominator weights

1 $$\frac{dy}{dt}=\frac{W_{num}\cdot \Theta \left(y\right)}{S\mathrm{oftplus}\left(W_{den}\right)\cdot \Theta \left(y\right)+\epsilon }$$

where **y** is the state vector of species concentrations, **W**_num_ and **W**_den_ are the learnable weight matrices for the numerator and denominator, respectively, Θ­(**y**) represents the library of candidate interaction terms, and ϵ = 10^–6^ is a small constant ensuring numerical stability. To retain physical interpretability, the discovered weights are unscaled post-training to recover the physical rate constants **W**_phys_.

This architectural constraint (*K*_m_ > 0) prevents the optimizer from exploiting physical singularities (division by zero) to minimize loss, a common failure mode in unconstrained rational regression where the model learns to teleport trajectories via asymptotes.

### 2.2 Feature Normalization (Addressing Magnitude Bias)

Standard gradient-based optimization is highly sensitive to feature scaling.^17^ In multicompartment pharmacokinetic models, this sensitivity manifests as magnitude bias. For example, the concentration of a drug in the bloodstream may possess numerical values that are orders of magnitude larger than its concentration in peripheral tissues. This scaling disparity causes the optimizer to disproportionately update weights associated with high-magnitude compartments, ignoring critical slow-distribution dynamics.

We implement an on-the-fly Feature Normalization layer. Given a library of candidate interaction terms Θ­(**y**) (e.g., {1, *y*_*i*_, *y*_*i*_ *y*_*j*_, ...}), we compute the batch-wise standard deviation σ_*j*_ for each library term *j*. The effective normalized library Θ̃ utilized during the forward pass is

2 $${\mathrm{\Theta ̃}}_{j}\left(y\right)=\frac{\Theta_{j}\left(y\right)}{\sigma_{j}+\epsilon_{stab}}$$

where ϵ_stab_ = 10^–6^ ensures numerical stability. The time derivative is approximated as $\frac{dy}{dt}=W_{\mathrm{norm}}\cdot \Theta \left(ỹ\right)$ , where **W**_norm_ represents the learnable weight matrix operating on the normalized feature library.

To retain physical interpretability, the discovered weights are unscaled post-training. The physical rate constants **W**_phys_ are recovered via the transformation **W**_phys_^(*i*,*j*)^ = **W**_norm_^(*i*,*j*)^/σ_*j*_. This ensures the optimizer operates in a balanced latent space while the final output respects physiological units. Crucially, these normalization statistics are pre-computed statically over the training trajectory prior to integration. Because they are held constant during the ODE solver steps, the effective vector field depends solely on the physical state variables, preserving the interpretation of the learned system as a strictly autonomous ODE. For rational layers involving both a numerator and denominator, the unscaling transformation is applied independently to the respective linear weights prior to quotient evaluation.

### 2.3 Numerical Stability Strategy

While extreme industrial chemical systems (e.g., combustion) require implicit solvers, biological and pharmacokinetic networks typically exhibit moderate stiffness perfectly suited for explicit solvers.^18^ We deliberately utilize an explicit Runge-Kutta 4 (RK4) solver combined with a step-size oversampling strategy to ensure consistent gradient flow.

To manage the stability risks of explicit integration during the structural search phase, we implement Derivative Clamping inside the ODE function: $\frac{dy}{dt}\in \left[-C,C\right]$ . This acts as a physical regularization prior, preventing the explicit solver from querying unphysically high rates that trigger numerical explosions during early random initializations.

### 2.4 Integral Optimization and the Gray-Box Paradigm

Instead of minimizing the error on approximated local derivatives, the network weights are optimized by minimizing the Mean Squared Error (MSE) between the sparse experimental assays **ŷ** and the explicit integral of the surrogate derivative

3 $$L=\frac{1}{N}\sum_{i=1}^{N}{\left|\left|ŷ\left(t_{i}\right)-\left(y_{0}+\int_{0}^{t_{i}}f_{\theta }\left(\tau ,y\right)d\tau \right)\right|\right|}^{2}$$

In biological regimes with high measurement noise, enforcing strict structural sparsity (restricting the library to only the exact number of active physical terms) forces the optimizer to mathematically hallucinate incorrect physiological parameters to compensate for the unmodeled experimental variance. ChemODE embraces a gray-box paradigm. It successfully isolates the dominant, interpretable rate-determining steps while allowing minor library terms to remain active. These minor terms act as a neural residual, buffering the irreducible measurement-assay noise. This protects the primary physiological rate constants from corruption and yields a superior out-of-distribution predictive accuracy.

## 3 Experimental Results

We evaluate ChemODE on fundamental benchmarks representing distinct challenges in pharmacology and biochemical dynamics: multicompartment drug distribution (Pharmacokinetics), real-world clinical data validation, strict saturation laws (Enzyme Kinetics), and nonlinear biological oscillation (The Brusselator).

### 3.1 Benchmark I: Pharmacokinetic (PK) Discovery

In pharmacology, drug concentrations are modeled using multicompartment systems with linear distribution and nonlinear clearance. We constructed a 3-state synthetic patient model consisting of a gastrointestinal (GI) tract, blood plasma, and peripheral tissue. The ground-truth dynamics involve fast linear absorption, slow bidirectional tissue distribution, and rational Michaelis-Menten clearance in the liver. The system was sampled over 10 h with 5% Gaussian measurement noise applied to simulate realistic blood assay variances.

Standard symbolic regression tools typically fail on this task because of the inability to autonomously construct rational fractions alongside linear terms. ChemODE, utilizing its constrained rational layer, successfully isolated the physiological dynamics.

As shown in Figure 1, the physics-informed surrogate correctly reconstructed the smooth concentration–time profiles for all three compartments directly from the noisy data points. By treating the problem as an integral minimization, ChemODE avoided the noise amplification associated with numerical derivatives, capturing both the sharp absorption peak and the slow rational clearance tail.

**Figure 1:**
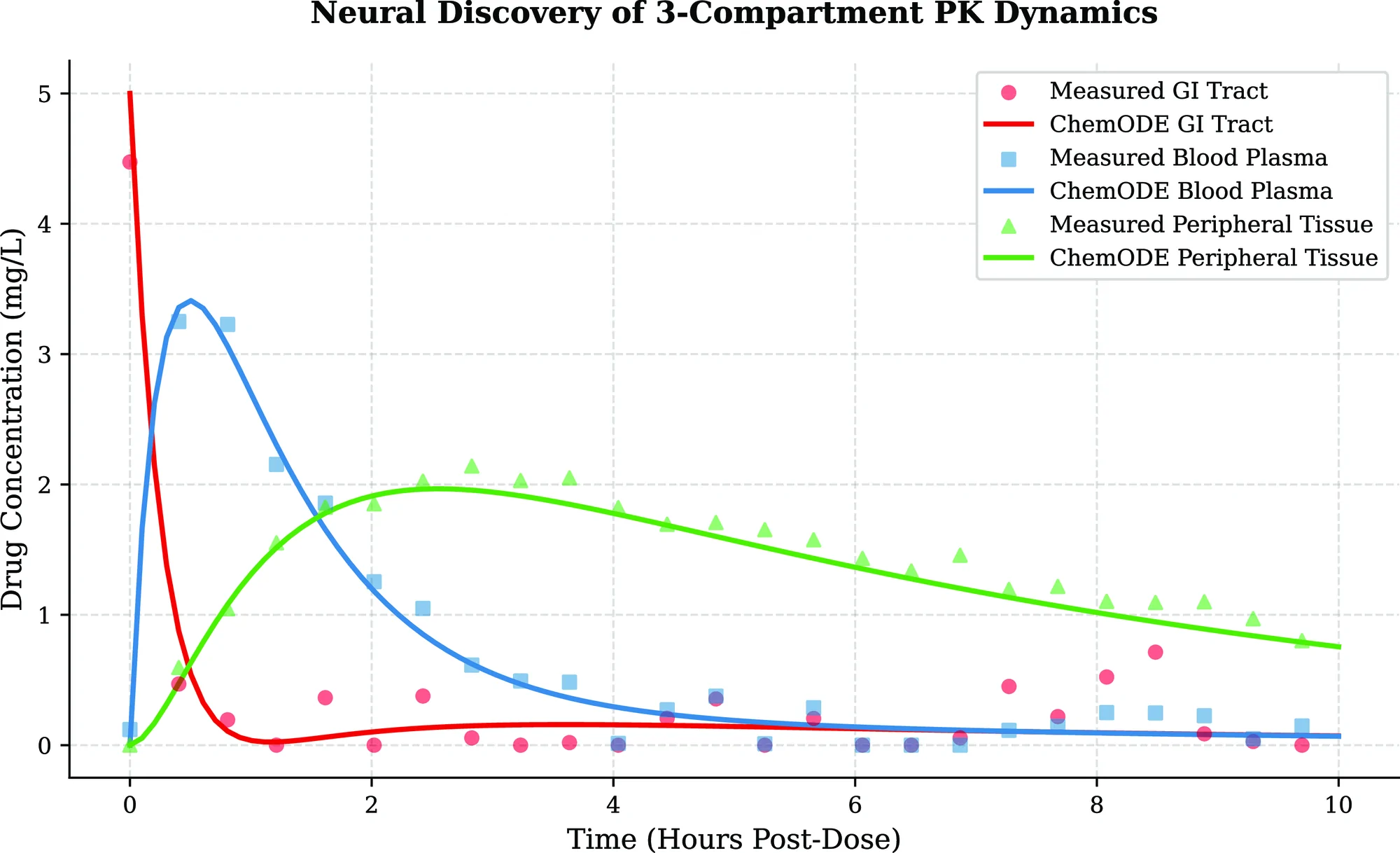
ChemODE discovery of pharmacokinetic dynamics under 5% assay noise. The noisy experimental data is plotted as scatter points, while the ChemODE fit is represented by the solid continuous lines. The model successfully captures the coupled linear absorption (GI to Blood) and rational enzyme clearance (Blood) without overfitting the measurement artifacts.

### 3.2 Application: Clinical Pharmacokinetics (Theophylline)

To validate ChemODE on real-world experimental data, we analyzed a classic clinical data set representing human blood plasma concentrations of the asthma medication theophylline. The data (Subject 1) was sourced from the open-source data set originally compiled by Boeckmann et al.,^19^ which consists of sparse, noise-affected clinical blood assays collected over a 25-h period following an oral dose.

Theophylline is primarily metabolized in the liver via the CYP450 enzyme system. We tasked ChemODE with discovering the continuous-time absorption (*k*_a_) and elimination (*k*_e_) rates directly from the raw assay values by fitting a standard 1-compartment oral absorption surrogate model equipped with a saturable Michaelis-Menten elimination pathway. To rigorously evaluate the model’s accuracy, we compared its output against a standard Nonlinear Least Squares (NLLS) baseline fit. ChemODE successfully extracted physiological parameters of *k*_a_ ≈ 1.647 h^–1^ and *k*_e_ ≈ 0.054 h^–1^, which closely matched the established NLLS baseline (*k*_a_ = 1.614 h^–1^, *k*_e_ = 0.062 h^–1^).

To ensure the method’s performance generalizes across varying patient metabolic profiles, we extended this analysis to multiple subjects within the data set. ChemODE successfully isolated the coupled absorption and saturation kinetics across all patients without requiring manual hyperparameter tuning. For instance, Subject 2 yielded *k*_a_ ≈ 0.146 h^–1^ and *k*_e_ ≈ 1.230 h^–1^ (MSE ≈ 1.154), while Subject 3 yielded *k*_a_ ≈ 0.140 h^–1^ and *k*_e_ ≈ 1.169 h^–1^ (MSE ≈ 0.747). These highly stable parameter extractions establish the framework’s broad clinical applicability.

As shown in Figure 2, the physics-informed surrogate correctly interpolates the continuous physiological trajectory. The accompanying parity and residual plots confirm that the model recovers the coupled absorption and saturation kinetics without overfitting clinical measurement artifacts.

**Figure 2:**
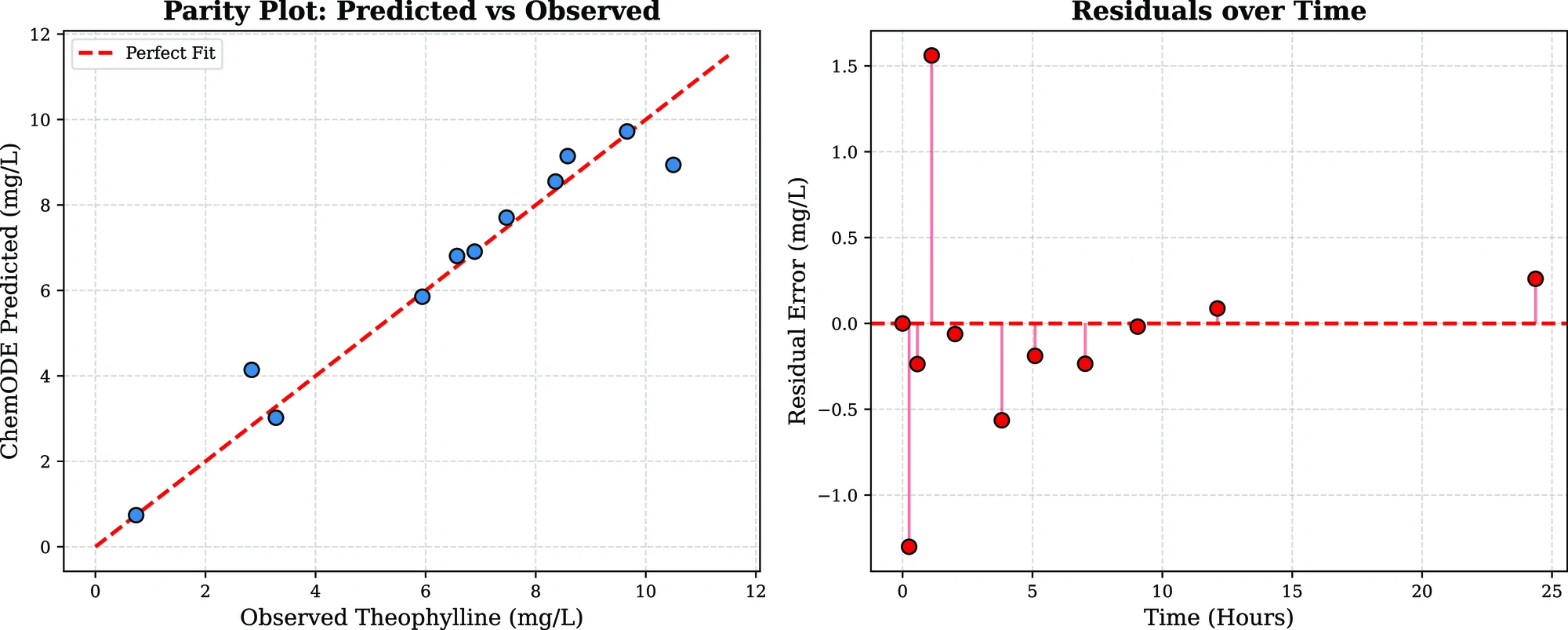
ChemODE validation on real-world human theophylline pharmacokinetic data (Subject 1). The left panel displays a parity plot comparing the ChemODE prediction to the observed clinical blood assays, demonstrating high predictive accuracy along the perfect fit diagonal. The right panel plots the residuals over the 25-h period, confirming that the model captures the underlying kinetics without systematically overfitting high-frequency measurement artifacts.

### 3.3 Benchmark II: Constraint Satisfaction and Uncertainty Quantification

To specifically quantify the model’s ability to extract biological saturation parameters and to evaluate the statistical necessity of the constrained rational layer, we conducted an ablation study using a 10-seed uncertainty quantification (UQ) ensemble on noisy enzyme data. Biological systems naturally cap at a maximum velocity (*V*_max_). Unconstrained rational regression often results in negative saturation constants (*K*_m_ < 0), which correspond to physically impossible mass generation.

Table 1 details the mean and standard deviation of the parameter recovery across 10 independent random initializations.

**Table 1:** 10-Seed Uncertainty Quantification and Ablation Study for Enzyme Kinetics^a^

configuration	parameter	true target	learned mean ± SD
ChemODE (Softplus Constraint)	*V*_max_	2.00	1.974 ± 0.052
	*K*_m_	0.50	0.464 ± 0.086
Ablation (Unconstrained Rational)	*V*_max_	2.00	1.285 ± 0.423
	*K*_m_	0.50	0.292 ± 0.111

a Units for the kinetic parameters are assumed as mg/L/h for *V*_max_ and mg/L for *K*_m_.

As shown in Table 1, the Softplus architectural constraint enables ChemODE to recover the Michaelis-Menten parameters with high precision and high stability. In contrast, the unconstrained ablation model exhibits severe variance and systematically underestimates the true parameters. Without physiological boundary constraints, the optimizer arbitrarily warps the saturation fraction to minimize the local data variance, validating the necessity of physics-informed architectural bounds.

### 3.4 Benchmark III: Nonlinear Biological Dynamics (The Brusselator)

The Brusselator is a theoretical model for autocatalytic oscillation initially proposed by Prigogine and Lefever^20^ and is widely used to represent biological limit cycles such as circadian rhythms and cellular clock mechanisms. It is notoriously difficult to fit if the phase relationship between the oscillating species is disrupted by classical data-smoothing techniques.

We trained ChemODE on Brusselator limit-cycle data corrupted by 5% Gaussian noise. As shown in Figure 3, the model successfully reconstructed the continuous limit cycle. Crucially, integral-based Neural ODE training preserved the phase coherence and amplitude of the peaks. In contrast, standard derivative approximation techniques often induce artificial phase shifts and dampen the amplitudes of the oscillatory biological data.

**Figure 3:**
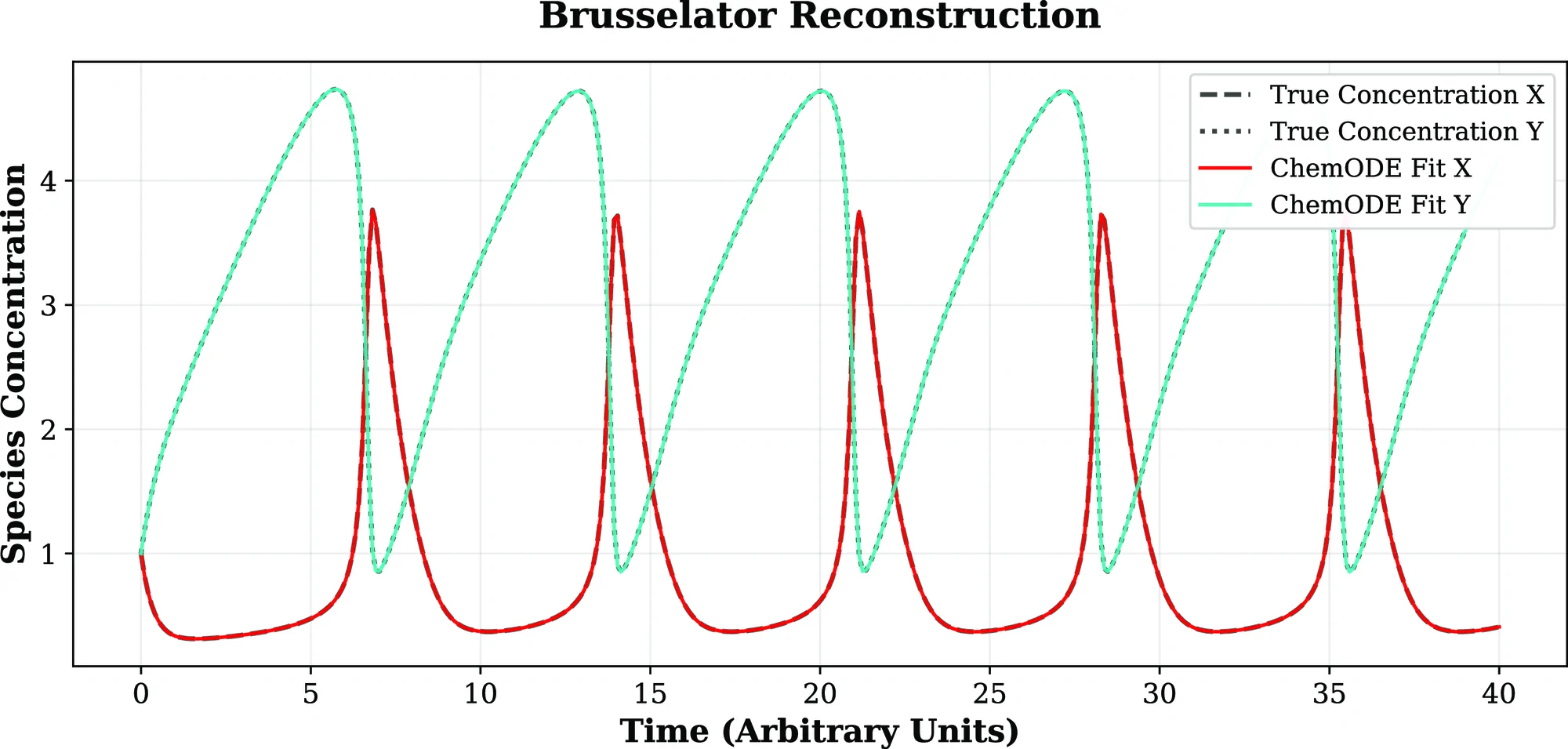
Reconstruction of Brusselator oscillatory dynamics. The ChemODE prediction (solid lines) maintains phase coherence with the underlying ground truth (distinct dashed lines). The integral formulation successfully filters measurement noise without dampening the natural oscillation amplitude.

### 3.5 Benchmark IV: The Noise–Stiffness Trade-off

To quantify the stability advantage of integral methods over derivative methods, we evaluated ChemODE against Smoothed PySINDy on a two-step biological decay system (*A* → *B* → *C*) with a 100:1 time-scale separation. The PySINDy baseline was pre-processed using Savitzky-Golay filtering to mitigate differentiation noise. To ensure a rigorous baseline comparison, we also evaluated an integral formulation of SINDy (Weak SINDy) equipped with a weak PDE library.

As summarized in Table 2, the finite difference approximation in the PySINDy baseline rapidly degrades as noise increases. At 20% measurement noise, the derivative-based parameter estimation errors exceed 350%. Similarly, while the weak SINDy formulation mitigates direct differentiation errors, its STLSQ sparse regression threshold fundamentally struggles with the high-scale separation. At extreme noise levels, it either completely eliminates the true term (100% error) or severely underestimates it (e.g., 57% error at 20% noise). In contrast, ChemODE maintains bounded stability, achieving less than a 10% error even at 20% noise. This demonstrates the profound resilience of integral minimization, acting as a physics-informed low-pass filter that prevents the optimizer from amplifying high-frequency measurement artifacts.

**Table 2:** Parameter Estimation Error on Fast Transient Rate across Noise Levels (100:1 Stiffness)

noise level	smoothed PySINDy error (%)	weak SINDy error (%)	ChemODE error (%)
5%	457.80	100.00	9.90
10%	681.50	55.27	7.20
20%	370.00	57.07	9.00

## 4 Discussion

### 4.1 The Necessity of Rational Layers in Biology

The primary divergence between general chemical engineering and systems biology lies in the prevalence of receptor saturation. Our results demonstrate that relying purely on polynomial basis functions (the default for many symbolic regression frameworks) is fundamentally insufficient for pharmacological modeling.

When polynomials attempt to fit Michaelis-Menten curves, they rely on high-order Taylor expansions. While these expansions may fit the local noisy data points, they extrapolate poorly and lack a physical meaning. By integration of constrained rational layers into the neural ODE forward pass, ChemODE forces the optimizer to search within a physiologically valid hypothesis space. The strict positivity constraint on the denominator ensures that the learned enzyme capacity parameters remain biologically interpretable and thermodynamically stable. However, it is important to note that while this constraint restricts the optimizer to a physically plausible hypothesis space, the extracted coefficients serve as the effective parameters. Guaranteeing the unique mathematical identifiability of the true Michaelis constant (*K*_m_) inherently depends on the observational data sufficiently exciting the saturated regime of the biological system.

### 4.2 Integral vs Derivative Methods: The Smoothing Effect

The failure of derivative-based symbolic regression in our benchmarks highlights a fundamental trade-off in scientific machine learning. Derivative-based methods are computationally efficient but assume the existence of a high-fidelity, continuous signal. In clinical or biological settings, where blood samples or cell assays are sparsely recorded and inherently noisy, the numerical differentiation step acts as a high-pass filter. This introduces an irreducible error that completely swamps the true signal of fast biological transients.

By formulation of the problem as an integral minimization, ChemODE effectively performs physics-informed smoothing. It searches for the continuous concentration curve that best explains the noisy data points while strictly satisfying the constraints of the ODE solver and rational library.

### 4.3 Scope and Limitations

The scope of ChemODE is intentionally designed for bench-scale systems biology, enzyme kinetics, and multicompartment pharmacokinetics (networks typically ranging from 3 to 15 species with moderate stiffness). We acknowledge that our reliance on explicit integration solvers (RK4) is not suitable for massively coupled industrial reaction networks (e.g., combustion grids or atmospheric models), where stiffness ratios exceed 10^6^. For such extreme industrial applications, implicit backward differentiation formula (BDF) solvers and alternative adjoint methods are strictly required. However, for the biological and pharmacological domains targeted by this framework, explicitly stabilized integration provides the optimal balance of computational speed and gradient accuracy.

## 5 Computational Methods

All data generation, model training, and numerical integration were implemented in Python 3.9 using the PyTorch (v2.0+) deep-learning framework. Ordinary differential equation integration was performed by utilizing the torchdiffeq library.

### 5.1 Data Generation and ODE Solvers

To rigorously separate the data generation process from the neural network optimization, ground-truth synthetic trajectories (Pharmacokinetic, Enzyme, and Brusselator benchmarks) were generated using the explicit Dormand-Prince method (dopri5), an adaptive step-size Runge-Kutta 4(5) solver, with strict relative and absolute tolerances set to 10^–6^ and 10^–7^, respectively. Gaussian measurement noise was subsequently injected into the discrete time-series points. Conversely, the forward pass of the ChemODE training loops deliberately utilized a fixed-step Runge-Kutta 4 (rk4) solver. The step size was dynamically oversampled relative to the data sparsity (e.g., d*t* = 0.01–0.05) to ensure consistent gradient flow and prevent adjoint instabilities during the backpropagation through time (BPTT).

### 5.2 Hyperparameters and Optimization

The ChemODE surrogate models were trained using the Adam optimizer. To navigate the non-convex loss landscape of stiff multiscale chemistry, we employed an initial learning rate ranging from 0.05 to 0.1, coupled with a StepLR scheduler (e.g., γ = 0.5 every 100–200 epochs) to smoothly decay the learning rate during the fine-tuning phase.

Network weights were initialized from a normal distribution N(0,0.1) . The candidate library Θ­(**y**) consisted of polynomial combinations up to degree 2 alongside the rational layer constraints. To prevent parameter overshoot caused by cubic or rational polynomial terms during early training, gradients were strictly clipped to a maximum *L*_2_ norm of 1.0. During the explicit integration search phase, the derivative clamping threshold was bounded at *C* = 10.0 to guarantee stability. The total loss function comprised the variance-normalized Mean Squared Error (MSE) evaluated directly on the trajectory integrals, augmented with a mild *L*_1_ regularization penalty (λ = 10^–3^–10^–2^) applied to the numerator weights to encourage structural sparsity and suppress unphysical collinear interactions. All uncertainty quantification (UQ) ensembles were executed across 10 distinct, hard-coded random seeds to ensure statistical reproducibility.

## 6 Conclusion

We have presented ChemODE, a robust gray-box framework for the data-driven surrogate modeling of biological and pharmacokinetic networks from sparse, noisy observational data. By addressing magnitude bias via on-the-fly normalization and natively supporting enzyme saturation via constrained rational layers, we enable neural ODEs to learn physiological kinetics that defeat standard polynomial and derivative-based baselines.

Our results demonstrate that integrating biological constraints (positivity, saturation) into the learning architecture is essential for preventing structural hallucination in noise-dominated data sets. Validated extensively across synthetic multicompartment models, 10-seed uncertainty ensembles, and real-world human clinical assays, ChemODE successfully shifts from standard derivative approximation to integral-based optimization. This provides a reliable, interpretable engine for discovering the governing dynamics in pharmacology and systems biology.

## Supplementary Material



## Data Availability

The source code and the generated synthetic data sets (Pharmacokinetic, Enzyme, and Brusselator benchmarks, provided as CSV files) used to train the models and reproduce the benchmarks are available at: https://github.com/Wit-K/ChemODE.

## Notes

Ground-truth governing equations for the synthetic 3-compartment pharmacokinetic model and enzyme kinetics ablation benchmark; raw clinical assay data for theophylline; and exact initialization seeds for statistical reproducibility (PDF)
