# Supplementary material for: ChemODE: A Physics-Informed Neural Surrogate for Robust Pharmacokinetics and Biochemical Dynamics
Source: ACS Omega. 2026 Sep 30;11(40):60914–9. doi: 10.1021/acsomega.6c04487 (PMC13670822; doi:10.1021/acsomega.6c04487)
Supplement: Supplementary file 1 [file ao6c04487_si_001.pdf]

Supporting Information for:

ChemODE: A Physics-Informed Neural Surrogate for Robust Pharmacokinetics and Biochemical Dynamics

Wit Kulvutiroj*
Kamnoetvidya Science Academy, Pa Yup Nai, Wang Chan District, Rayong 21210, Thailand

*Corresponding Author Email: wit.kulvutiroj@gmail.com

S1 Ground Truth Governing Equations

To generate the synthetic observational data for the benchmarks presented in the main manuscript, the following exact ordinary differential equations (ODEs) were integrated using the `dopri5` explicit solver.

S1.1 Synthetic 3-Compartment Pharmacokinetic (PK) Model

The synthetic pharmacokinetic dataset represents a patient receiving an oral dose of a drug, which distributes between the Gastrointestinal (GI) tract, Blood Plasma, and Peripheral Tissue. The liver clearance mechanism follows non-linear Michaelis-Menten saturation kinetics. The governing equations are defined as:

$$\frac{d[\mathrm{GI}]}{dt} = -k_a[\mathrm{GI}] \tag{S1}$$

$$\frac{d[\mathrm{Blood}]}{dt} = k_a[\mathrm{GI}] - k_{12}[\mathrm{Blood}] + k_{21}[\mathrm{Tissue}] - \frac{V_{max}[\mathrm{Blood}]}{K_m + [\mathrm{Blood}]} \tag{S2}$$

$$\frac{d[\mathrm{Tissue}]}{dt} = k_{12}[\mathrm{Blood}] - k_{21}[\mathrm{Tissue}] \tag{S3}$$

The true physiological parameters utilized for data generation were: Absorption rate $k_a = 5.0$, distribution rate to tissue $k_{12} = 0.5$, redistribution rate to blood $k_{21} = 0.2$, maximum clearance velocity $V_{max} = 1.0$, and Michaelis constant $K_m = 0.5$. The system was simulated over 10 hours with initial conditions $[2.0, 0.0, 0.0]$, $[5.0, 0.0, 0.0]$, and $[1.0, 0.0, 0.0]$ representing three distinct dosage profiles.

S1.2 Enzyme Kinetics Ablation Benchmark

For the 10-seed uncertainty quantification and architectural ablation study, an isolated target representing a purely saturating enzymatic decay was generated:

$$\frac{dy}{dt} = -\frac{V_{max} \cdot y}{K_m + y} \tag{S4}$$

The true kinetic parameters were set to $V_{max} = 2.0$ and $K_m = 0.5$, with an initial substrate concentration of $y_0 = 5.0$.

S2 Real-World Clinical Dataset: Theophylline

To validate ChemODE on real-world experimental assays, we utilized a publicly available clinical dataset detailing the pharmacokinetics of the anti-asthmatic drug Theophylline. The data represents sparse blood plasma concentrations collected from human Patient #1 following an oral administration at $t = 0$. The raw numerical values used to train the neural surrogate are provided in Table S1.

Table S1: Raw Clinical Assay Data for Theophylline (Patient #1).

**Time (Hours)**	**Blood Plasma Concentration (mg/L)**
0.00	0.00
0.25	2.84
0.57	6.57
1.12	10.50
2.02	8.33
3.82	5.80
5.10	5.09
7.03	3.95
9.05	2.53
12.12	1.47
24.37	0.00

S3 Statistical Reproducibility and Initialization Seeds

To ensure complete statistical rigor and reproducibility in the evaluation of the Constrained Rational Layer, the uncertainty quantification (UQ) ablation study reported in the main manuscript was executed using ten independent PyTorch initialization seeds. These distinct starting states ensure that the reported mean and standard deviation accurately reflect the optimizer's global convergence behavior rather than a localized initialization artifact.

The exact random seeds utilized for the PyTorch and NumPy pseudo-random number generators during the 10-trial enzyme kinetics ensemble were: **42, 12, 99, 1024, 2026, 7, 777, 888, 123, 456**.
